# Long term use of eltrombopag in children with chronic immune thrombocytopenia: extended real life retrospective multicenter experience of the Italian Association of Pediatric Hematology and Oncology

**DOI:** 10.3389/fmed.2023.1214308

**Published:** 2023-07-14

**Authors:** Paola Giordano, Giuseppe Lassandro, Angelica Barone, Simone Cesaro, Ilaria Fotzi, Fiorina Giona, Chiara Gorio, Angela Maggio, Maurizio Miano, Antonio Marzollo, Margherita Nardi, Andrea Pession, Antonio Ruggiero, Giovanna Russo, Paola Saracco, Marco Spinelli, Alessandra Tolva, Assunta Tornesello, Valentina Palladino, Giovanni Carlo Del Vecchio

**Affiliations:** ^1^Interdisciplinary Department of Medicine, Pediatric Unit “B. Trambusti”, University of Bari Aldo Moro, Bari, Italy; ^2^Department of Pediatric Onco-Hematology, University Hospital of Parma, Parma, Italy; ^3^Pediatric Hematology Oncology, Department of Mother and Child, Azienda Ospedaliera Universitaria Integrata, Verona, Italy; ^4^Department Pediatric Hematology Oncology, Azienda Ospedaliero Universitaria A. Meyer Children Hospital, Florence, Italy; ^5^Department of Translational and Precision Medicine, Sapienza University of Rome, Rome, Italy; ^6^Hemato-Oncology Unit, Children Hospital, Spedali Civili, Brescia, Italy; ^7^Department of Hematology, IRCCS Casa Sollievo della Sofferenza, San Giovanni Rotondo, Italy; ^8^Clinical and Experimental Hematology Unit, “G. Gaslini” Children's Hospital, Genoa, Italy; ^9^Pediatric Hematology-Oncology Unit, Department of Women's and Children's Health, Azienda Ospedaliera-University of Padova, Padua, Italy; ^10^Pediatric Hematology, Azienda Ospedaliero Universitaria Pisana, Pisa, Italy; ^11^Department of Pediatrics, Sant'Orsola-Malpighi Hospital, University of Bologna, Bologna, Italy; ^12^Pediatric Oncology Unit, Fondazione Policlinico Universitario A. Gemelli IRCCS, Università Cattolica del Sacro Cuore, Roma, Italy; ^13^Pediatric Hemato-Oncology Unit, Department of Clinical and Experimental Medicine, University of Catania, Catania, Italy; ^14^Pediatric Hematology, Department of Pediatrics, University Hospital Città della Salute e della Scienza, Turin, Italy; ^15^Clinica Pediatrica, Fondazione IRCCS San Gerardo dei Tintori, Monza, Italy; ^16^Pediatric Hematology/Oncology, IRCCS Policlinico San Matteo Foundation, University of Pavia, Pavia, Italy; ^17^Pediatric Hematology Oncology, Presidio Ospedaliero Vito Fazzi, Lecce, Italy

**Keywords:** immune thrombocytopenia, eltrombopag, children, thrombopoietin receptor agonists, bleeding disorders

## Abstract

**Background:**

The present multicenter retrospective study on eltrombopag administration in Italian children with chronic ITP aims to extend follow-up of our previous study.

**Materials and methods:**

This retrospective multicenter study was conducted in 17 centers affiliated to the Italian Association of Pediatric Hematology and Oncology (AIEOP). Patients were classified into three subgroups: group 1 included patients who discontinued treatment due to a stable platelet count; group 2 included patients who discontinued treatment due to ineffectiveness; group 3 included patients who did not permanently discontinue treatment.

**Results:**

56 patients were eligible for analysis. The median duration of eltrombopag treatment was 40 months (7–71 months). Twenty patients (36%) discontinued permanently eltrombopag. The reasons of permanent discontinuation were adverse effects (*n* = 1), inefficacy (*n* = 10), stable platelet count (*n* = 9). All patients of group 1 maintained a durable response without additional treatments after eltrombopag discontinuation. We found that patients of group 2 were on treatment for less time (median treatment time: 13.5 months, min: 6.0 – max: 56.0) than patients of group 1 (median treatment time: 34 months, min: 16.0 – max: 62.0) (*p* < 0.05). Patients of group 2 mostly did not achieve a stable platelet count in the first 6 months of treatment and underwent concomitant therapies during follow-up respect of group 1 and group 3 (*p* < 0.01).

**Conclusion:**

Our study found that the benefits of eltrombopag treatment, in terms of platelet count improvement and use of additional therapies, are identifiable from the first 6 months of treatment.

## Introduction

Immune thrombocytopenia (ITP) is the most common acquired bleeding disorder in children characterized by a low platelet count (<100 × 10^9^/L) in the absence of underlying causes. ITP spontaneous remission rate is elevated in childhood. Approximately, 20% of children develop chronic ITP defined as an immune thrombocytopenia that lasts more than 12 months (1–5). Thrombocytopenia in ITP is mostly due to increase platelet destruction caused by autoreactive antibodies that bind platelets targeting them for phagocytosis by macrophages residing in the spleen and liver. In addition, B-cell hyperreactivity, T-cell–mediated cytotoxicity and impaired megakaryocytes central maturation contribute to the perpetuation of the autoimmune process (6, 7).

Usually, corticosteroids and intravenous immunoglobulins have used as first-line treatments in children. Secondary options include thrombopoietin receptor agonists (TPO-RAs), immunosuppressive drugs (rituximab, mycophenolatemofetil, and sirolimus) or splenectomy (1, 2, 8).

Eltrombopag is an orally bio-available non peptide TPO-RAs that interacts with the transmembrane domain of the thrombopoietin receptor leading to activation of the JAK/STAT pathway and increases megakaryocytopoiesis and platelets production (9). Eltrombopag has been shown to be safe and effective for children with ITP both in clinical trials and in clinical practice. Two international, randomized, double-blind, placebo-controlled trials (PETIT 1 and PETIT 2), reported a response rate to eltrombopag ranged from 62 to 75%, with a good treatment tolerability (10, 11). Recently, our study group of the Italian Association of Pediatric Hematology and Oncology (AIEOP) have firstly conducted a retrospective multicenter study with the objectives to determine in Italian current clinical practice the prevalence of eltrombopag use children affected by chronic ITP and to assess efficacy and safety outside clinical trials. We have demonstrated that in Italian clinical pediatric practice eltrombopag was widely used, effective in improve platelet count in the first 6 months of treatment and safe during the whole period of follow-up (12).

Although several studies have subsequently confirmed the efficacy and tolerability of eltrombopag administration in children (13–15), to date studies evaluating long-term follow-up and treatment discontinuation in pediatric patients are limited (14). Moreover, the frequency and potential predictive factors (16) of durable response in children after eltrombopag withdrawal are largely unknown.

The present multicenter retrospective study on eltrombopag administration in Italian children with chronic ITP aims to extend follow-up of our previous study (12), to evaluate eltrombopag long-term follow-up data, focusing on the duration of eltrombopag treatment and its potential permanent discontinuation.

## Materials and methods

### Study cohort

This retrospective multicenter study was conducted in 17 centers affiliated to the Italian AIEOP between April 2016 and April 2022.

Patients with chronic ITP previously enrolled, aged 1–17 years, who were still on treatment after 6 months of follow-up were included in the analysis. Patients with non-immune or hereditary thrombocytopenia were excluded.

Data regarding eltrombopag first administration, age at onset of thrombocytopenia, gender, age at the start of eltrombopag, number of previous ITP therapies and baseline platelet count were obtained. Duration of treatment, dosage, platelet count, disease duration and concomitant medications were recorded. We recorded the adverse effects (AEs) that occurred after the last follow-up of the previous study. Patients were monitored closely for possible AEs evaluating monthly hematology assays and clinical chemistry.

Patients were classified into three subgroups: group 1 included patients who discontinued treatment due to a stable platelet count, group 2 included patients who discontinued treatment due to ineffectiveness, and group 3 included patients who did not permanently discontinue treatment.

### Study definitions

Chronic ITP was defined using the International Consensus Guidelines as a platelet count <100 × 10^9^/L that lasts 12 months or longer (5). The baseline platelet count was the pre-treatment value closest to the first dose of eltrombopag. Thrombocytosis was defined as a platelet count ≥450 × 10^9^/L (10). Increased transaminases were described for alanine aminotransferase (ALT) and aspartate aminotransferase (AST) >3× normal value according to the previous clinical trials (10, 11). Complete response (CR) and partial response (PR) were defined as a platelet count >100 × 10^9^/L and 30–100 × 10^9^/L, respectively (5, 13). Permanent discontinuation was considered for a stable platelet count defined as a platelet count>50 × 10^9^/L at ≥75% of assessments in the preceding 6 months (16) or for a no response (NR) defined as a platelet count less than 30 × 10^9^/L, or less than a two-fold increase in the baseline platelet count, or bleeding events after administration of an appropriate dose of eltrombopag for 12 weeks (5).

### Statisical analysis

Standard statistical methods were used to summarize the data: frequency and percentage for categorical variables, median (minimum and maximum) for continuous scaled variables.

To measure the proportion of patients remaining on eltrombopag treatment for a certain amount of time after start of treatment Kaplan–Meier estimate was used. To test whether the difference between survival times between two groups was significant log-rank test and Wilcoxon method was used.

Comparisons of clinical and laboratoristic characteristics among groups of patients were performed using nonparametric statistical tests: Wilcoxon rank-sum test (for comparing two groups) and Kruskal-wallis tests (for comparing more than two groups) for continuous variables and Fisher exact test for categorical variables.

Agreement between measurement of categorical variables was performed by Cohen’s kappa.

All calculated *p* values were 2 sided, and a level of 0.05 was used for assessing significance.

All analysis were conducted using SAS software (version 9.4).

## Results

### Demographic and baseline data

Of fifty-seven patients enrolled, 56 patients were eligible for analysis while 1 patient was excluded because of a diagnosis of Bernard Soulier Syndrome.

Demographic and baseline clinical data of patients included into the study are reported in Table 1.

**Table 1:** Demographic and baseline features of the included patients.

**Total**		
*n*	56	
**Gender, *n* (%)**		
M	22	(39%)
F	34	(61%)
**Age at onset of thrombocytopenia**, years		
Median (min–max)	9	(2–17)
**Baseline platelet count**, × 10^9/L		
Median (min–max)	10	(2–49)
**Age at the start of eltrombopag**, years		
Median (min–max)	13	(3–17)
**ITP duration before the first dose**, years		
Median (min–max)	2	(1–12)
**Eltrombopag starting dose,** mg		
Median (min–max)	25	(25–50)
**Second-line therapies prior eltrombopag**, *n* (%)		
**Yes**	31	(55%)
Cyclosporine	10	(23%)
Mycophenolatemofetil	21	(48%)
Rituximab	4	(9%)
Sirolimus	9	(20%)
**No**	25	(45%)

### Proportion of patients remaining on eltrombopag treatment and permanent discontinuation

The median duration of eltrombopag treatment was 40 months (7–71 months). The median platelet count at last follow-up was 78 × 10^9^/L (2–700 × 10^9^/L).

Twenty patients (36%) discontinued permanently eltrombopag. The reasons of permanent discontinuation were adverse effects (*n* = 1), inefficacy (*n* = 10), stable platelet count (*n* = 9).

**Figure 1:**
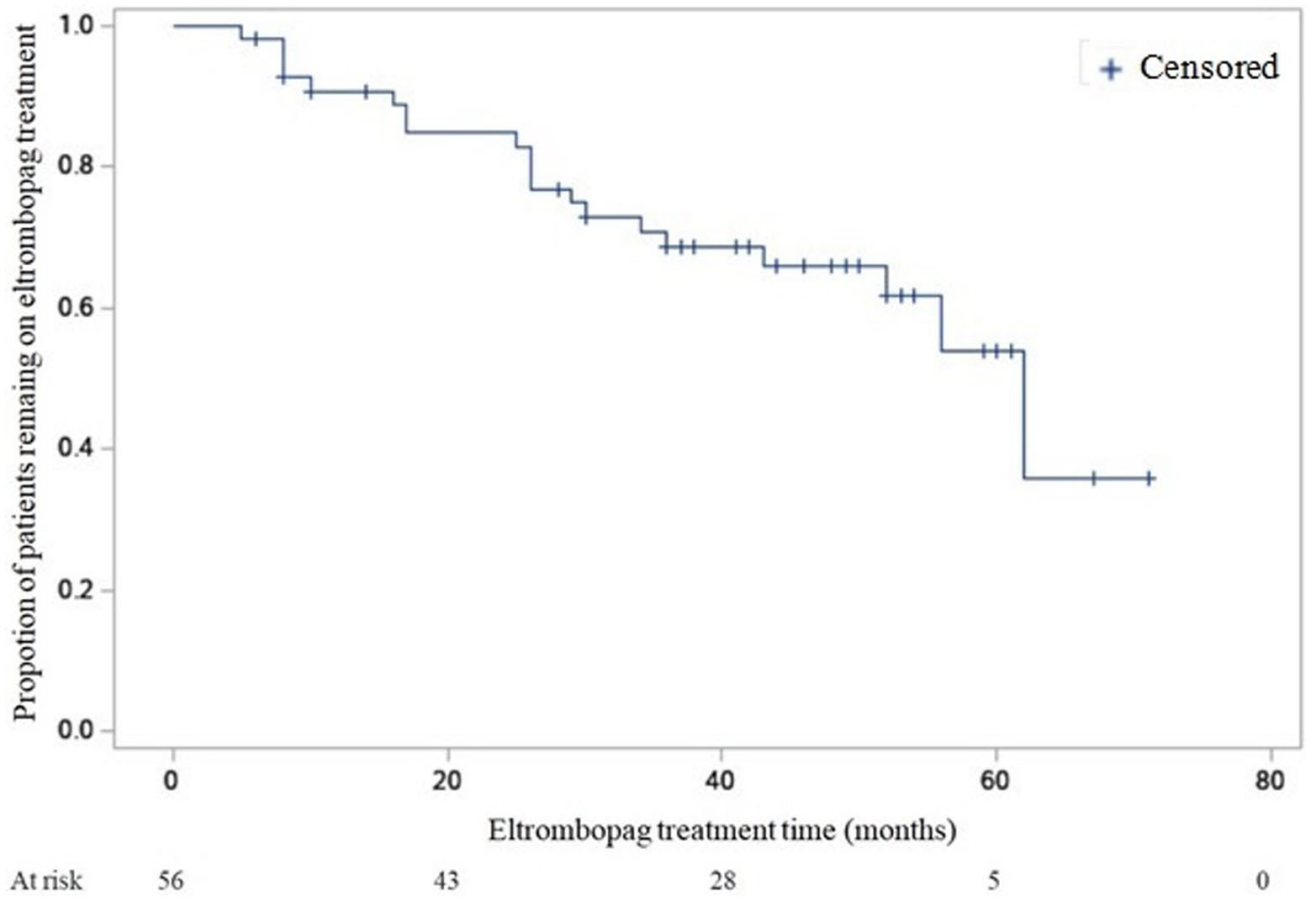
Kaplan–Meier estimation of the probability of remaining on eltrombopag treatment during follow-up.

A Kaplan–Meier estimation of the probability of remaining on eltrombopag treatment during follow-up is reported in Figure 1.

Difference evaluation between proportion of patients remaining on eltrombopag treatment between Group 1 and Group 2 gave the following result: log-rank test *p* = NS, Wilcoxon *p* < 0.05.

The median time of treatment was 34 months in group 1 (95% confidence interval [CI], 16–52 months) and 13.5 months in group 2 (95% CI, 6–26 months).

### Clinical and laboratoristic characteristics among groups of patients

Among patients that discontinued permanently eltrombopag due to a stable platelet count, 6 patients (67%) achieved a complete response while 3 patients (33%) achieved a platelet count ≥50 × 10^9^/l at the time of discontinuation of eltrombopag. Their median platelet count at last follow-up was 85 × 10^9^/l (77–171 × 10^9^/l). Subsequently, all patients of group 1 maintained a durable response without additional treatments after eltrombopag discontinuation (median time of follow-up 15 months, min: 6 – max 36 months).

Patients of group 2 were on treatment for less time (Median treatment time: 13.5 months, min: 6.0 – max: 56.0) than patients of group 1 (Median treatment time: 34 months, min: 16.0 – max: 62.0) (*p* < 0.05).

Age at the start of eltrombopag and ITP duration before eltrombopag in the 3 Groups are reported in Table 2, while concomitant therapies, and the number of patients with PLT > 50 × 10^9^/L at ≥75% of assessments in and after the first 6 months of therapy are reported in Table 3.

**Table 2:** Group 1: patients who discontinued treatment due to a stable platelet count; Group 2: patients who discontinued treatment due to ineffectiveness; Group 3: included patients who did not permanently discontinue treatment.

	Group 1	Group 2	Group 3	*p* value
**Age at the start of eltrombopag, months** (Median, min–max)	150.8 (37.0–190.3)	133.7 (78.7–170.6)	159.5 (33.1–214.2)	ns
**ITP duration before eltrombopag**,**months** (Median, min–max)	22.2 (12.4–113.9)	25.0 (13.3–62.6)	30.76 (12–147.4)	ns

ns, not significant.

**Table 3:** Group 1: patients who discontinued treatment due to a stable platelet count, Group 2: patients who discontinued treatment due to ineffectiveness, Group 3: included patients who did not permanently discontinue treatment.

	Group 1	Group 2	Group 3	*p* value
**Concomitant therapies**				
**Yes**	0	6	6	
**No**	9	4	30	<0.01
**Number of patients with PLT > 50 × 10**^**9**^**/L at ≥ 75% of assessments in the first 6 months of therapy**				
**Yes**	8	2	25	
**No**	1	8	11	<0.01
**Number of patients with PLT > 50 × 10**^**9**^**/L at ≥ 75% of assessments after the first 6 months of therapy**				
**Yes**	9	0	24	
**No**	0	10	12	<0.0001

ns, not significant.

Thirteen patients (23%) required one or more concomitant ITP medications during follow-up, mainly consisting of IVIG (*n* = 9). Other therapies included corticosteroids (*n* = 2), mycophenolate mofetil (*n* = 2), rituximab (*n* = 1), and sirolimus (*n* = 1).

**Figure 2:**
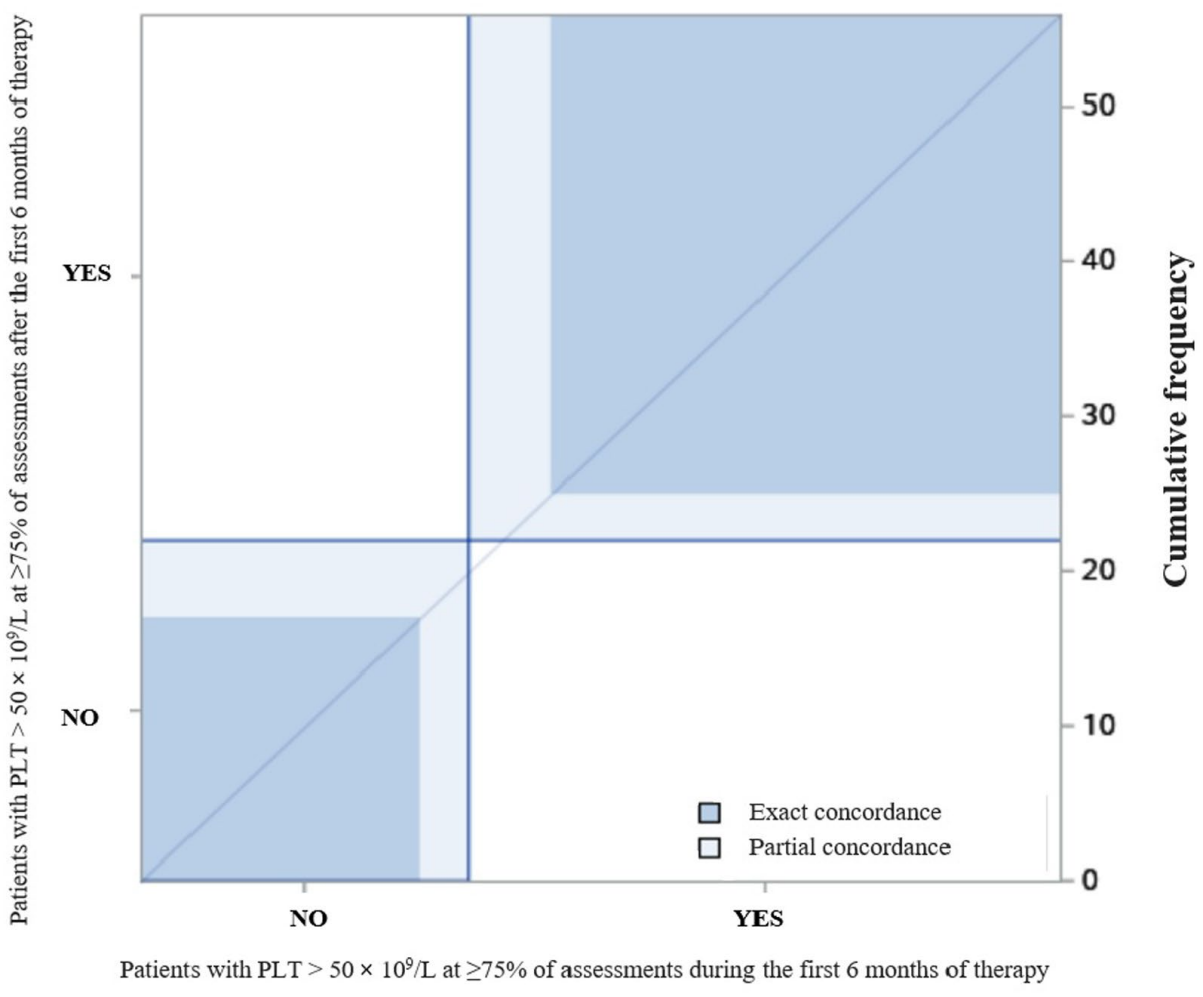
The degree of agreement between patients who had PLT > 50 × 10^9^/L at ≥75% of assessments during and after the first 6 months of therapy.

The degree of agreement between patients who had PLT > 50 × 10^9^/L at ≥75% of assessments in the first 6 months of therapy and patients who had PLT > 50 × 10^9^/L at ≥75% of assessments after the first 6 months of therapy is reported in Figure 2. The κ coefficient was 0.69 (95% confidence interval [CI]: 0.50–0.89).

### Adverse effects

The most frequent AEs were headache, thrombocytosis and microcytosis. Six patients (11%) developed one or more events of headache at a dosage ranging from 7 to 75 mg/day with a median platelet count of 145 × 10^9^/L (58–209 × 10^9^/L). Four patients (7%) experienced thrombocytosis at a dosage ranging from 50 to 75 mg/day. In all cases thrombocytosis resolved after short-term discontinuation of eltrombopag. Three patients (5%) developed iron-deficiency anaemia while 2 patients (4%) developed microcytosis in absence of anaemia. One patient (2%) aged 6 years while on eltrombopag (62.5 mg/day) underwent a transitory elevation of transaminases more than three times the normal value associated with vomiting, abdominal pain and jaundice after 41 months of treatment. The elevation of transaminases resolved after a temporary dose reduction of eltrombopag. One patient (2%) aged 17 years, while on eltrombopag (50 mg/day) underwent after 39 months of treatment a mild transitory elevation of transaminases less than three times the normal value, in the absence of either clinical symptoms or increased direct bilirubin. The elevation of transaminases resolved after a temporary dose reduction of eltrombopag. One patient (2%) aged 15 years with heterozygous Factor V Leiden mutation who had experienced cerebral venous thrombosis life-threatening during eltrombopag treatment as reported in previous study, after temporary interruption of drug resumed treatment and developed after 39 months of treatment deep vein thrombosis of the lower limbs (eltrombopag dosage of 75 mg/day) (12). Other transitory AEs were oral aphthous ulcers (2%), allergic urticaria (2%), diarrhea (2%) and persistent asthenia (2%).

## Discussion

Eltrombopag is an orally bioavailable non peptide thrombopoietin receptor agonist, approved by the USA Food and Drug Administration (FDA) in 2015 for pediatric patients with chronic ITP failing first-line therapies, and by the European Medicines Agency (EMA) in 2016. Recently, eltrombopag use was extended for pediatric patients with ITP that lasts longer than 6 months. The efficacy and safety of eltrombopag in pediatric ITP has been widely reported in the literature. In PETIT1 and PETIT2 pediatric trials has been demonstrated that eltrombopag improves platelet counts with a response rate ranged from 62 to 75%, reduces the use of concomitant ITP therapies with limited adverse effects (10, 11). Our previous real-life study conducted in Italy on 386 children with chronic ITP showed that 19% of patients were treated with eltrombopag and of these, 55% did not require ITP rescue therapies during the first 6 months of eltrombopag treatment. Moreover, the median platelet counts and proportion of children achieving the target platelet count of at least 30 × 10^9^/L and 100 × 10^9^/L significantly increased during the first 6 months of therapy (12). In 2020 Cheng et al., confirmed that eltrombopag was safe, well tolerated, and effective in maintaining platelet count and reducing bleeding in children affected by persistent and chronic ITP and treated with eltrombopag for up to 12 months (15). Although numerous studies have been conducted on the administration of eltrombopag in children with ITP, they have a limited follow-up period. Furthermore, studies evaluating permanent discontinuation are scarce. Yozgat et al. described the treatment outcome of eltrombopag in children with ITP for up to 43 months of treatment (mean duration of treatment: 12.3 ± 10 months, range: 1–43 months). In this study 75% of patients were still on treatment at their last follow-up while eltrombopag treatment was stopped in 25% of patients. The reasons for discontinuation were lack of response (66%), non-adherence (15%), adverse effects (8%), response to treatment (11%).

In our present study we extended follow-up description of patients enrolled in our previous study (12) for up to 71 months.

Similarly to Yozgat et al. (14), we found that at their last follow-up 65% of the patients were still in treatment and mostly with a sustainable platelet count while 35% of patients discontinued eltrombopag treatment. Of patients who permanently discontinued eltrombopag, half of patients discontinued treatment due to ineffectiveness. An equivalent number of patients discontinued treatment due to stable platelet counts.

Compared to data from Yozgat et al. (14), in our study we found a higher percentage of patients who discontinued treatment because they achieved a stable platelet count. None of these patients experienced disease relapse after therapy discontinuation over a median follow-up of 15 months.

Similar results were reported in adults (17, 18). Mahe’vas et al. found, in accordance to our results, that a substantial proportion of patients receiving TPO-RAs maintain a durable response following discontinuation (19). In 2019 Gonzalez-Lopez et al. found that 38 adult ITP patients (51.3%) maintained treatment-free response 36 months after stopping eltrombopag with no need of additional ITP therapies (20). Cervinek et al. reported that all patients treated with TPO-RAs maintained remission following therapy discontinuation over a median follow-up of 33 months (21).

To date, the pathophysiological mechanisms responsible for a durable response following eltrombopag withdrawal remains largely unknown. Recently, Ghadaki et al., hypothesized that eltrombopag use may rebalance platelet immunological tolerance similarly to the induction of immune tolerance by administering high doses of factor VIII concentrate in haemophilic patients with inhibitor (22–24). It has also been described that eltrombopag improves T and B regulatory cells functions, reduce T cells and monocytes activation (25).

Several questions remain regarding the identification of suitable patients for successful discontinuation. Recently, a consensus statement on the use of TPO-RAs in adult patients affected by ITP identified patient characteristics that may be potential indicators of remission or relapse during tapering or discontinuation of TPO-RAs. These factors included age, ITP duration, platelet count, duration of therapy, dosage of treatment and use of additional therapies (16). According to the ITP experts, we compared patients who discontinued treatment due to a stable platelet count, patients who discontinued treatment due to ineffectiveness and patients who did not permanently discontinue treatment with the aims to identify predictive factors of successful discontinuation. Testing for differences in survivor fuctions between treatment duration of patients, we have found that patients who discontinued treatment due to ineffectiveness were on treatment for less time than patients who discontinued treatment due to adequate platelet count. Only Wilcoxon statistic was significant and not log-rank test, probably because Wilcoxon test gives more weight to early times than to late times. Moreover, the difference in duration was evident comparing patients who discontinued treatment due to a stable platelet count and patients who discontinued treatment due to ineffectiveness.

We found that the age at the start of eltrombopag and ITP duration before eltrombopag did not influence treatment response in terms of discontinuation. Moreover, we found that patients who discontinued treatment due to ineffectiveness mostly did not achieve a stable platelet count in the first 6 months of treatment and underwent concomitant therapies during follow-up respect patients who discontinued treatment due to a stable platelet count and patients who did not permanently discontinue treatment. In addition, we found that patients who achieved adequate platelet counts in the first 6 months of treatment were more likely to maintain them after the first 6 months of treatment.

Our study suggests that some children may not require eltrombopag indefinitely. Moreover, treatment discontinuation is worth considering in patients with ITP who achieved adequate platelet counts in the first 6 months of treatment and did not need additional treatments for ITP. In addition, the no-response to eltrombopag in the first 6 months of treatment requires consideration of therapeutic alternatives.

The main limitation of this study is that it is a retrospective study. Further prospective studies including a larger number of children are required to confirm our findings and to research additional predictive factors of durable response after eltrombopag discontinuation.

In our study, long-term eltrombopag use appeared to be well tolerated, with adverse effects in accordance with its safety profile. We found that the most common adverse events were headache and thrombocytosis as previous studies have reported (10–13). These adverse effects were transient and resolved after eltrombopag dosage reduction. Nine percent of patients developed microcytosis accompanied to normal or low haemoglobin value. Several studies conducted *in vitro* and *in vivo* have demonstrated that eltrombopag has iron chelation properties (14). Punzo et al., suggested the possible therapeutic application of eltrombopag in the treatment of iron overload in patients with thalassemia major in combination with other iron chelating drugs (26). Moreover, eltrombopag administration appears useful in inhibiting human cytomegalovirus replication by iron chelation (27). Subsequently, two clinical studies found that eltrombopag administration reduced mean corpuscular volume and ferritin levels in children with ITP (14, 28). Our results are similar to previous studies. We suggest that haemoglobin, mean corpuscular volume, and ferritin levels should be periodically assessed in children treated with eltrombopag.

In our study we reported that one patient aged 15 years with heterozygous Factor V Leiden mutation experienced cerebral venous thrombosis and subsequently deep vein thrombosis of lower limbs at eltrombopag dosage of 75 mg/day. Both venous and arterial thrombotic events were described in eltrombopag adult trials (29, 30).

Additionally, several studies reported that patients with ITP have an increased risk for thromboembolic complications. Many predisposing factors to thrombosis such as the presence of antiphospholipid antibodies and platelet microparticles derived from platelet membranes, were identified in ITP. In addition, the use of corticosteroids and IVIG may increase risk for arterial and venous thrombosis (31–34). Although ITP may represent a risk factor for thrombotic events, it has been described that TPO-RAs administration in ITP patients may contribute to thrombogenicity (35), and monitoring for these complications is essential. A recent study suggested that patients treated with eltrombopag may have an increase in the microparticle-associated phosphatidylserine procoagulant activity and in PAI-1 levels (36). However, the pathogenetic mechanism that increases the thrombophilic risk during eltrombopag therapy is not well established. In this study we reported one patient with heterozygous Factor V Leiden mutation who had experienced cerebral venous thrombosis during eltrombopag treatment and, after a temporary interruption of drug, he resumed treatment and developed deep vein thrombosis of the lower limbs. The Italian Medicines Agency does not provide indications regarding dose interruption or modification in case of thrombotic events during eltrombopag treatment.^1^ The risk–benefit ratio of discontinuing eltrombopag should be weighed considering platelet count, risk of bleeding and potential use of alternative therapies. In most patients who have experienced a thrombotic episode, eltrombopag treatment is discontinued (37). However, the potential efficacy of restart eltrombopag therapy after a thrombotic episode has been reported (38, 39). Thrombophilic screening is not recommended in childhood but it may be useful to screen children treated with eltrombopag and with a family history of thrombotic events (40). Moreover, after a first episode of thrombosis, the opportunity to resume eltrombopag treatment should be evaluated on a case-by-case basis and further studies should be conducted to provide more evidence.

In conclusion, the use of eltrombopag in childhood could offer a good chance of definitively treating ITP, with long-term side effects comparable to those reported in previous studies. Our study found that the benefits of eltrombopag treatment, in terms of platelet count improvement and use of additional therapies, are identifiable from the first 6 months of treatment. However, identifying additional potential predictors of remission requires further studies.

## Data availability statement

The original contributions presented in the study are included in the article/supplementary material, further inquiries can be directed to the corresponding author.

## Ethics statement

The studies involving human participants were reviewed and approved by Comitato Etico Locale Policlinico di Bari. Written informed consent to participate in this study was provided by the participants’ legal guardian/next of kin.

## Author contributions

PG and GL contributed because together conceived the project, coordinated the working group, and wrote the draft version. VP and GD performed the statistical analysis. All authors contributed to the article and approved the submitted version.

## Conflict of interest

The authors declare that the research was conducted in the absence of any commercial or financial relationships that could be construed as a potential conflict of interest.

## Publisher’s note

All claims expressed in this article are solely those of the authors and do not necessarily represent those of their affiliated organizations, or those of the publisher, the editors and the reviewers. Any product that may be evaluated in this article, or claim that may be made by its manufacturer, is not guaranteed or endorsed by the publisher.

## Footnotes

^1^https://farmaci.agenziafarmaco.gov.it

## Abbreviations

ITP: Immune thrombocytopenia
AIEOP: Italian Association of Pediatric Hematology and Oncology
TPO-RAs: Thrombopoietin receptor agonists
AEs: Adverse effects
ALT: Alanine aminotransferase
AST: Aspartate aminotransferase
CR: Complete response
PR: Partial response
NR: No response
PAI-1: Plasminogen activator inhibitor-1
